# Understanding hydrodynamic wear in self-similar superhydrophobic coatings subjected to rapid droplet impacts†

**DOI:** 10.1039/d3ra00700f

**Published:** 2023-04-19

**Authors:** Daniel J. Braconnier, Terence Davidovits, Randall M. Erb

**Affiliations:** a Department of Mechanical and Industrial Engineering, Northeastern University 360 Huntington Avenue Boston MA 02115 USA r.erb@northeastern.edu; b Thermobionics LLC Boston MA USA

## Abstract

Superhydrophobic materials rely on both chemical apolarity and surface roughness to achieve the high contact angles and the low roll-off angles that lead to self-cleaning and antibacterial properties. Current superhydrophobic coatings tend to be delicate and lose their properties easily when subjected to droplet impact. Such impact deteriorates these coatings through hydrodynamic wear; changing structure, eroding hydrophobic chemistry, and quickly leading to full wet out of the substrate. In fact, hydrodynamic wear is more detrimental to coatings than seemingly more aggressive mechanical wear including scratching with sandpaper – a common approach used to claim both self-similarity of a material and extreme robustness against wear. What makes certain coatings more robust against hydrodynamic wear? To understand this answer, we systematically study ten disparate self-similar superhydrophobic coating approaches from academia to industry by subjecting them to hydrodynamic wear with rapid droplet impacts. We offer an iteration of a spinning disk methodology that enables parallel testing of multiple coatings simultaneously. We have developed an analytical model that accurately estimates the average size and velocity of droplets created from the spinning disk. We find rapid droplet impacts that simulate a medium rain can deteriorate most coatings within seconds or minutes, with certain exceptions lasting up to 22 days. The more resilient coatings share common attributes including robust apolar chemistry, hierarchal topography, and a slow loss of sacrificial material. The best performing coatings can be characterized using power-law relationships that parallel mechanical fatigue functions and provide a predictive quantitative metric for the performance of hydrophobic coatings. Overall, this paper offers a quantitative approach to hydrodynamic wear of self-similar superhydrophobic coatings.

## Abstract 2

Rapid droplet impact testing has been leveraged to identify the most resilient self-similar superhydrophobic materials exhibit apolar chemistry, hierarchical topography, and gradual spallation.

## Graphical abstract

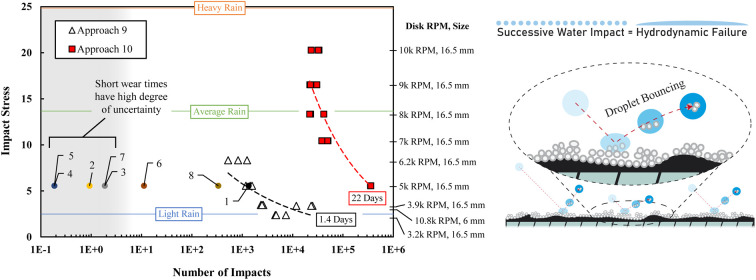

## 1 Introduction

Superhydrophobic materials offer compelling advantages to create self-cleaning, antibacterial, and water repellant materials that have far reaching applications from solar cells to radomes to next generation textiles.^1–3^ Almost exclusively, superhydrophobic materials leverage the combination of hierarchical roughness and chemical apolarity to achieve high contact angles and low roll-off angles.^1,4,5^ Hierarchical roughness often comes from a combination of micron-sized and nano-sized particulates, while apolar chemistry is supplied by a hydrophobic matrix or surface chemistry.^1,5,6^ Images of water beading on or bouncing off superhydrophobic materials instill a feeling of immediacy to these applications being realized, but several challenges remain that have so far limited real-world usage.^7,8^

One key remaining challenge is that the majority of reported superhydrophobic materials are incredibly fragile, being easily disrupted with a finger's touch.^5,7^ To improve coating robustness, an idea that is gaining popularity is to leverage self-similar materials that, when mechanically worn, continually expose a similar surface as the original superhydrophobic surface.^6,9–16^ A goal of this approach is to greatly increase the lifetime of a coating subjected to real-world conditions such as light to heavy rain conditions.

**Fig. 1:**
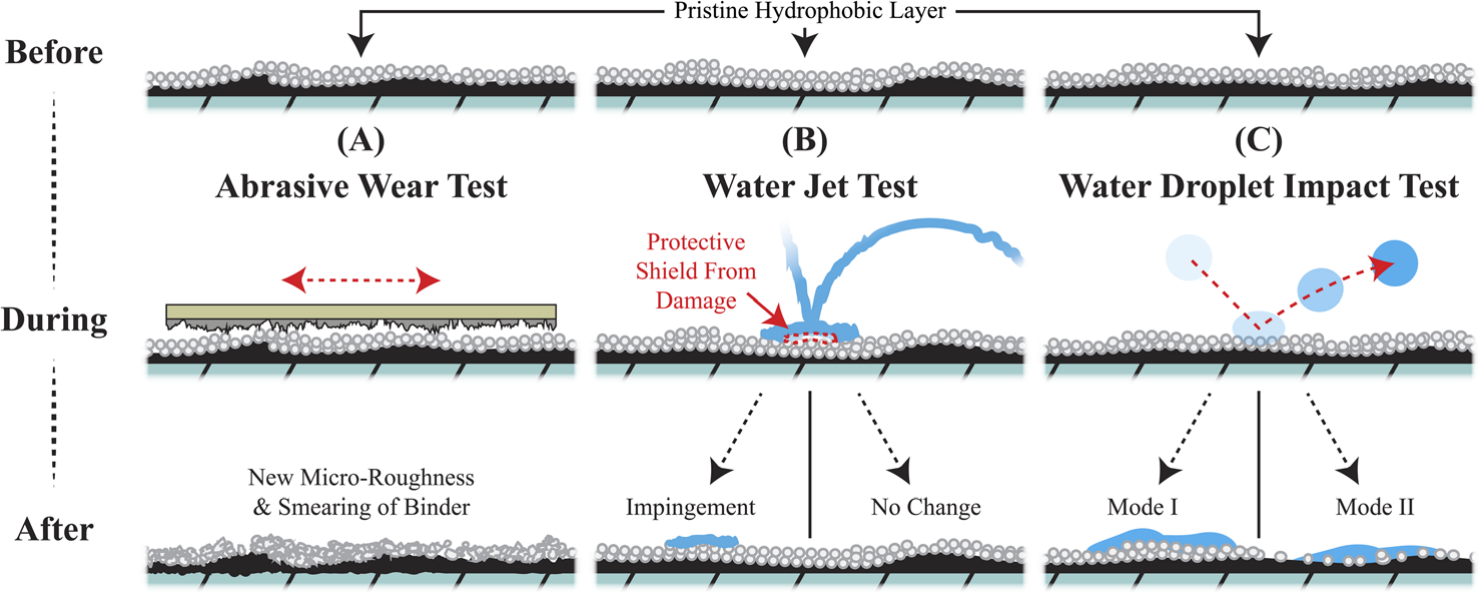
(A) Abrasive wear testing smears and roughens the coating. Depending on the binder material, this test can increase the coatings overall hierarchal roughness and, thus, can even increase the coating's contact angle. (B) Water jet testing is a good test to determine the coating's critical stress for water impingement, but the continuous nature provides a protective shield that can artificially preserve the coating material's integrity. (C) Water droplet impact testing uses discrete droplets to measure the coating material's impingement resistance and fatigue resistance in a manner consistent with application-based water impact like rain.

The success of these self-similar materials is commonly demonstrated by a set of wear tests followed by contact angle measurements.^8^ The three most common wear tests for hydrophobic coatings are visualized below in Fig. 1. First, abrasive wear testing, *via* sandpaper, is a popular test to show the general robustness of a superhydrophobic coating.^6,9–21^ Sandpaper abrasive wear testing damages the coating and will leave behind a smeared and highly roughened surface which sometimes exhibits contact angle measurements with little deviation from the pristine coating.^17–19^ Second, water jet testing subjects coatings to a constant stream of water which can be visually dramatic.^20,21^ In actuality, the continuous stream of water creates an entrapped layer of air at the impact site that can serve as a shield to the coating.^15,17,20–24^ Third, water droplet impact tests are conventionally conducted by dropping water at a constant rate from a specific height above the coating.^8,15,17,18,24^ With each impact, a large, localized stress is cyclically applied to the coating's surface as the kinetic energy of the droplet is absorbed leading to two modes of failure in the hydrophobic coating.^22,23,25^ Mode I failure involves pinning where droplets adhere to and wet-out the surface. Mode II failure involves coating removal where bouncing droplets physically remove hydrophobic material from the coating. Mode II wear will eventually result in mode I failure. Of the three tests, water droplet impact tests are the close proxy to rainfall and are surprisingly aggressive in deteriorating the hydrophobic nature of coatings relative to the other two tests. Understanding the onset and dependencies of the failure modes under water droplet impact testing may lead to superior hydrophobic coatings exhibiting rain repellency for a wide range of applications.

In this work, we explore the design principles of self-similar hydrophobic coatings that can withstand repeated droplet impacts. First, we offer a robust augmentation of droplet impact testing that we term Rapid Droplet Impact (RDI) testing. RDI testing allows for accelerated, parallelized, and facile testing of a diversity of coatings by leveraging a spinning disk to create small, fast-moving droplets with controllable trajectories. Second, we synthesize and subject ten diverse coating types to RDI testing to inform linkages between structure, property, and performance outcomes. Third, for the most robust coatings, we test across impact conditions that range from light to heavier rain equivalencies and develop a hydrodynamic wear model that can predict coating lifetimes. Finally, we offer a summary of apparent design principles that lead to the longest hydrophobic lifetimes. Formalization of the RDI testing approach and the application relevancy to rainfall should provide a framework for testing the performance of future coatings.

## 2 Rapid droplet impact (RDI) wear test framework

### 2.1 Implementation of RDI wear test framework

**Fig. 2:**
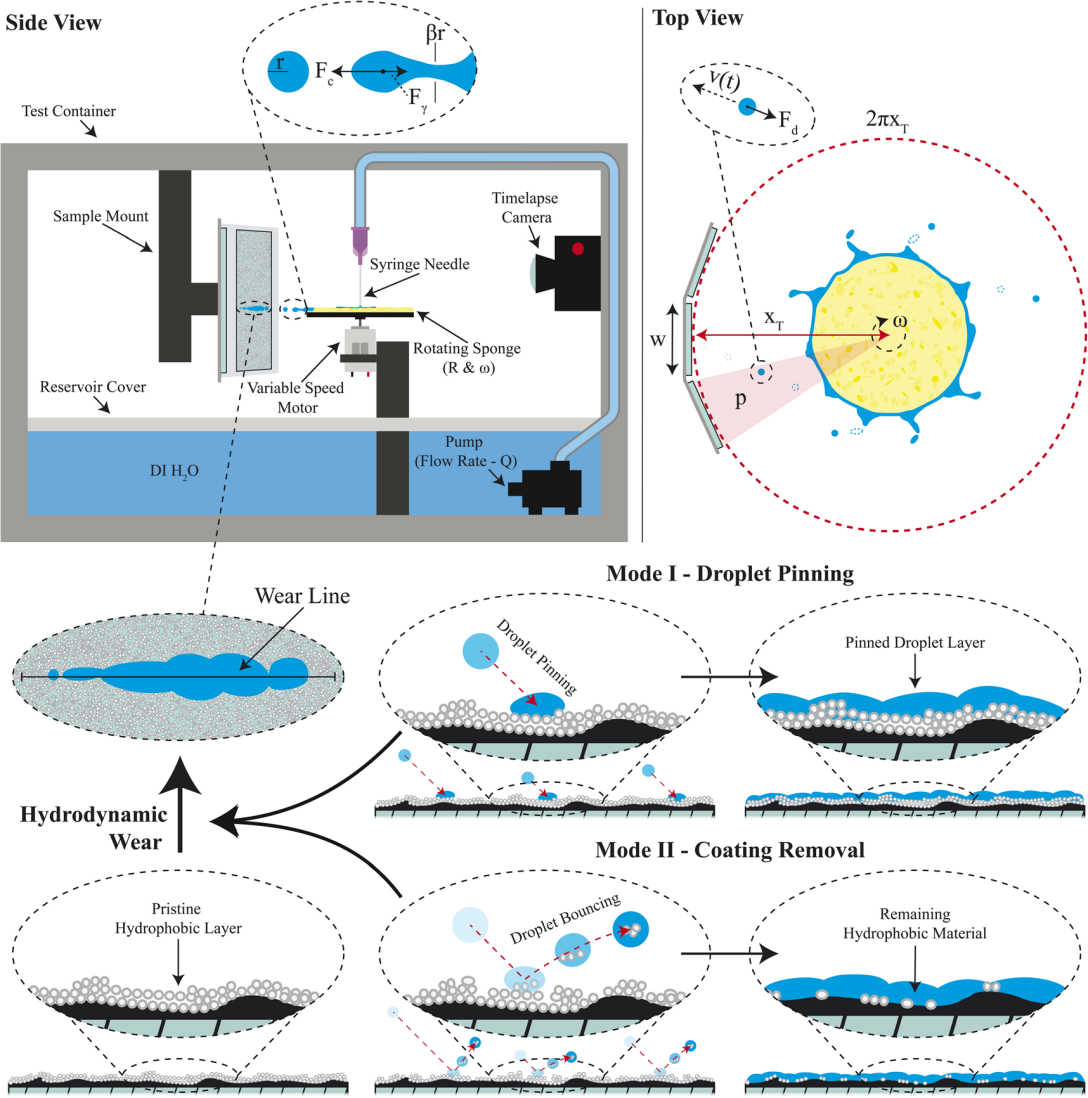
Proposed testing framework to subject multiple coated specimens to rapid droplet impacts. Water is fed through the syringe needle into a hydrophilic sponge-covered disk that continuously spins to generate controlled droplets. These droplets impact the coatings of mounted samples over time causing hydrodynamic wear *via* two modes of failure.

A custom test apparatus was fabricated to controllably apply rapid droplet impacts in the form of precise droplet sizes and velocities simultaneously onto multiple mounted samples with superhydrophobic coatings (Fig. 2). In addition to precise droplet control, this custom apparatus was inexpensive to build and suitable for long duration testing (even out to 22 days) of multiple samples simultaneously. The detailed specifications of the setup are provided in Section 6. Briefly, a variable speed motor horizontally rotated a disk mounted sponge at speeds ranging from 1000-rpm to 11 310-rpm. Disk sizes of 6 mm, 16.5 mm, 32 mm, and 62 mm were used to increase the angular velocity and droplet size range that was achievable. Onto this sponge, deionized water was dispensed through a syringe needle at a rate controlled by a water pump. This water would fully saturate the sponge. Then, the centrifugal force of the rotating disc would drive water off from the sponge edges in the form of droplets. The droplets would break off when the centrifugal force of the growing droplet eventually overcame the surface tension keeping the droplet attached to the hydrated sponge. These droplets would impact samples at velocities ranging from 0.8 to 10 m s^−1^. The samples were in the form of coated microscope slides, though the setup is amenable to other form factors. Droplet sizes and velocities were characterized using a high-speed camera to understand and validate the setup.

### 2.2 Analytic model of RDI wear test framework

The spinning disk has previously been leveraged to create controlled water droplets from rotary atomization.^26,27^ The predominant focus of previous work has been on higher viscosity fluids including low surface tension oils that generate ligaments and droplets under low-speed conditions (below 2000-rpm).^26–30^ The analytic models offered in these works do not describe well the lower viscosity and higher surface tension water droplets generated from a high rpm spinning sponge in the setup considered here. To understand the droplet size, velocity, and impact stress, we have developed a first principles analytic model that shows consistent prediction of the size of generated water droplets and their associated kinetic energy through the spinning disk method. Water is filled onto the top of the spinning disk at a set volumetric flow rate, *Q*, and spreads across the disk toward the edges. For the case of the hydrophilic sponge disk implemented in this work, the spreading is very homogeneous throughout the disk. As the disk spins, the centrifugal force drives the water outward from the disk, first forming fluidic fingering that thins into capillaries that eventually break causing droplets to fly tangentially away from the spinning disk (Fig. 2).

The centrifugal force, *F*_C_, on these droplets immediately before separation can be expressed as *F*_C_ = *ω*^2^*Rm*. Here *ω* is the angular velocity of the disk, *R* is the disk radius, and *m* is the mass of the droplet. Droplets will break away from the spinning disk when a critical mass of fluid has accrued at the end of the capillary to overcome the interfacial tension force of *F*_γ_ = *β*2π*rγ*, where, *γ* is the surface tension of the fluid in air, *r* is the droplet radius, and *β* is the ratio between the radius of the droplet and the radius of the created capillary as defined in Fig. 2. Of note, previous work has determined that *β* has complicated dependencies on the fluid and disk characteristics,^29,30^ but here we determined treating *β* as a simple constant was sufficient to still encompass the first principles of droplet creation within water-based systems.

The radius of a droplet created off a spinning disk was determined through the force balance of the centrifugal force and the interfacial tension force acting on a mass of fluid (*i.e.*, *F*_γ_ ≈ *F*_C_), allowing for a direct analytical prediction of the radius of the droplet as:1
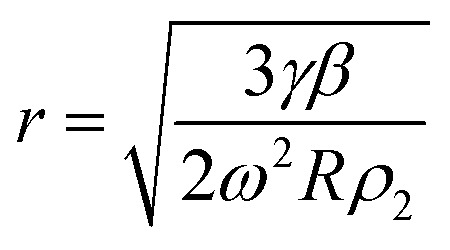
Here, the droplet is treated as a perfect sphere where the mass is *m* = *ρ*_2_(4π/3)*r*^3^ and *ρ*_2_ is the density of the fluid. Once the droplet has disconnected from the fluid left on the disk, it will fly tangentially outward from the disk. During flight, the fluid droplet is subjected to drag. Newtonian mechanics can be used to predict the resultant droplet velocity over time, *t*, when subjected to a drag force as:2
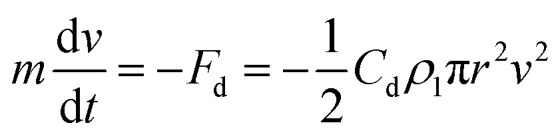
Here, *F*_d_ is the drag force for a spherical droplet, *C*_d_ is the drag coefficient, *ρ*_1_ is the density of air, and *v* is the traveling velocity of the droplet. The droplets seem slightly oblate during flight, but this does not significantly affect the outcome of the drag force (ESI†).

This first-order differential equation can be solved given the boundary conditions that at time *t* = 0 the droplet velocity would be equal to the tangential velocity (*v*_*t*=0_ = *ωR*) of the disk's edge. Therefore, the velocity of the droplet over time can be predicted as:3
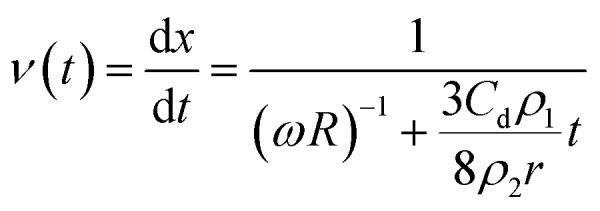


Eqn (3) can be used to solve for the droplet velocity across its flight path up to the point of impact (ESI†) resulting in an analytic expression for the impact velocity of a droplet, v*, as:4
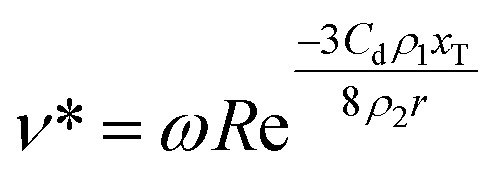


Therefore, the kinetic energy of single droplet at impact is defined as follows:5
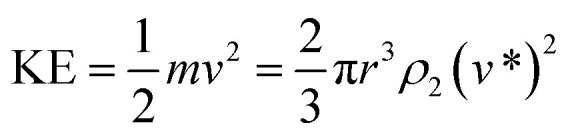


Droplets during impact are known to spread out on a surface.^22,23^ We make the rough assumption that the bulk of the kinetic energy is transferred across the original geometry of the water droplet with the spreading motion of the droplet accounting for a smaller portion of energy transfer and the droplet compressing nearly its full diameter. With such a simplification, the impact force of each droplet, *F*_I_, is defined as *F*_I_ ≈ KE/2*r* leading to a calculated stress applied to the coating by a droplet as follows:6
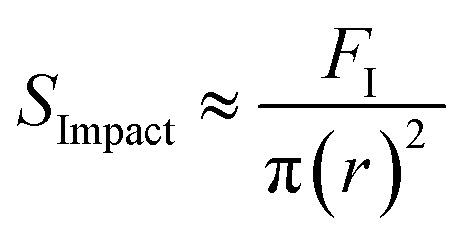


To validate this numerical model and gain confidence in the ability to control droplet generation, high speed videography was implemented. Specifically, a Chronos 1.4 high speed camera was used to capture the sizes and velocities of droplets created from disks ranging in size from 6 mm up to 62 mm and spinning at speeds from 1000-rpm up to 11 310-rpm. This camera enabled the measuring of droplets moving up to 10 m s^−1^ in speed. Faster droplets generated from higher rpm and larger disks were not considered.

**Fig. 3:**
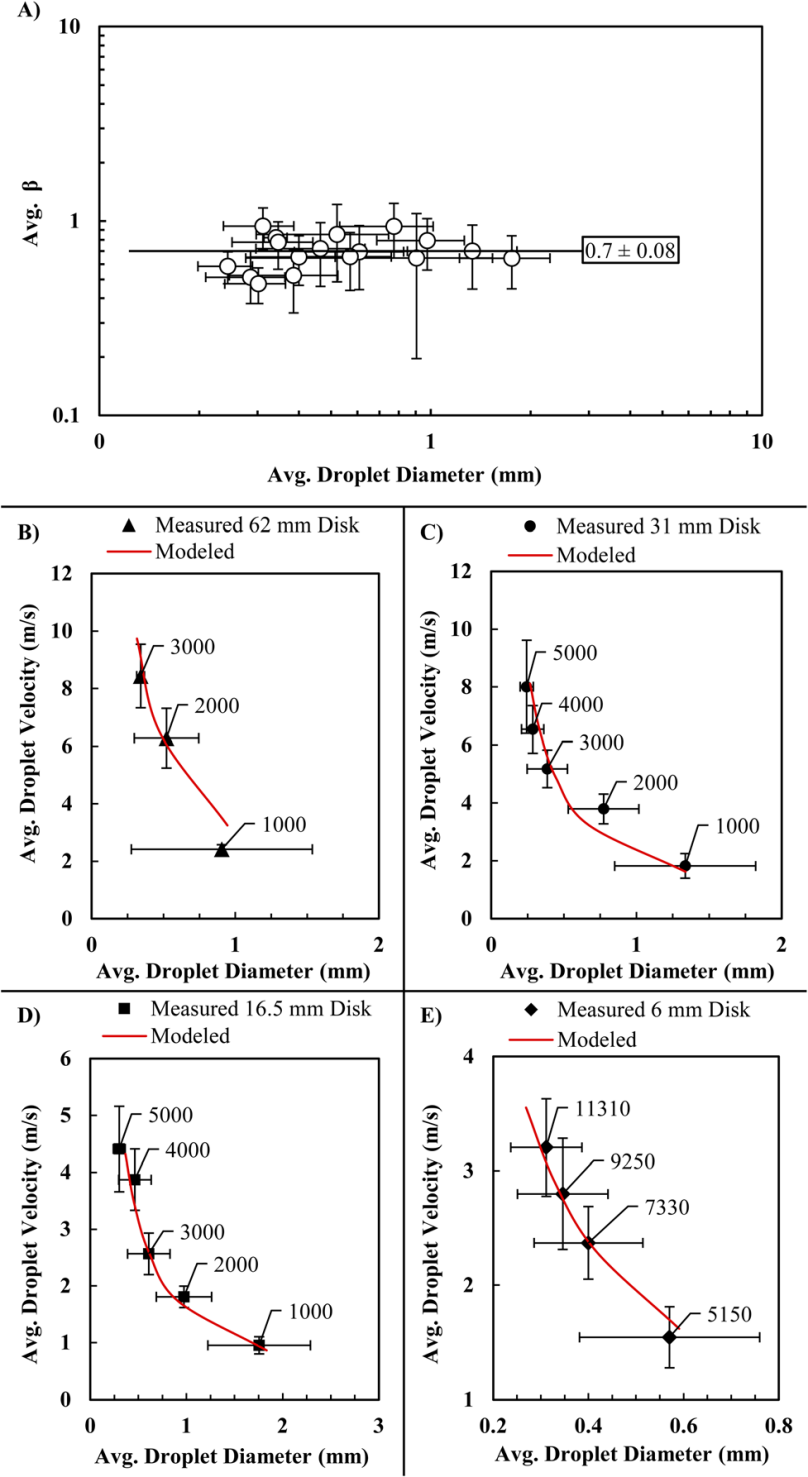
(A) Beta values per measured droplet diameters show that the value of beta changes little across different test conditions. (B–E) Droplet diameter model with an excellent fit to recorded droplet velocity and diameter values (disk rpm called out).

The parameter *β* was experimentally determined from recorded droplet diameter data and was found to consistently be ∼0.7 across experimental conditions (Fig. 3A). Measured droplet sizes and velocities were graphed and compared to modeled values across 17 different system conditions (Fig. 3B–E). As demonstrated, the simplified analytic model proved remarkably accurate for predicting the average size and velocity of water droplets generated from a spinning disk across diverse conditions. The larger standard deviations in certain cases should be noted and are attributed to defects in the sponge covering the disk, imperfect alignments in the setup resulting in slight wobble, and the effects of higher order non-equilibrium stochastics that effects fluid instabilities during droplet breakup. To reduce the impact of these larger standard deviations, the disk size of 16.5 mm, that demonstrated the most consistency, was used as the standard testing condition for assessing coating lifetimes *via* RDI. Further, this RDI testing level is associated with light to average rain conditions which are relevant in many applications (Fig. 5).

In addition to sizes and velocities, the effect of drag on droplets was assessed from video footage, which indicated that drag can be ignored for the short distances associated with the RDI testing apparatus (*C*_d_ ≈ 0 and v* = *ωR*, ESI†).

## 3 Synthesis and RDI testing of diverse approaches to self-similar superhydrophobic coatings

In this work, we created ten diverse superhydrophobic coatings that were tested with RDI. We provide the complete details, protocols, and experimental validation in ESI.† Below we report brief descriptions for each synthesis process, each contact angle measurement, and each RDI result. All wear results in this section are from our 16.5 mm disk at 5000-rpm test.

**Fig. 4:**
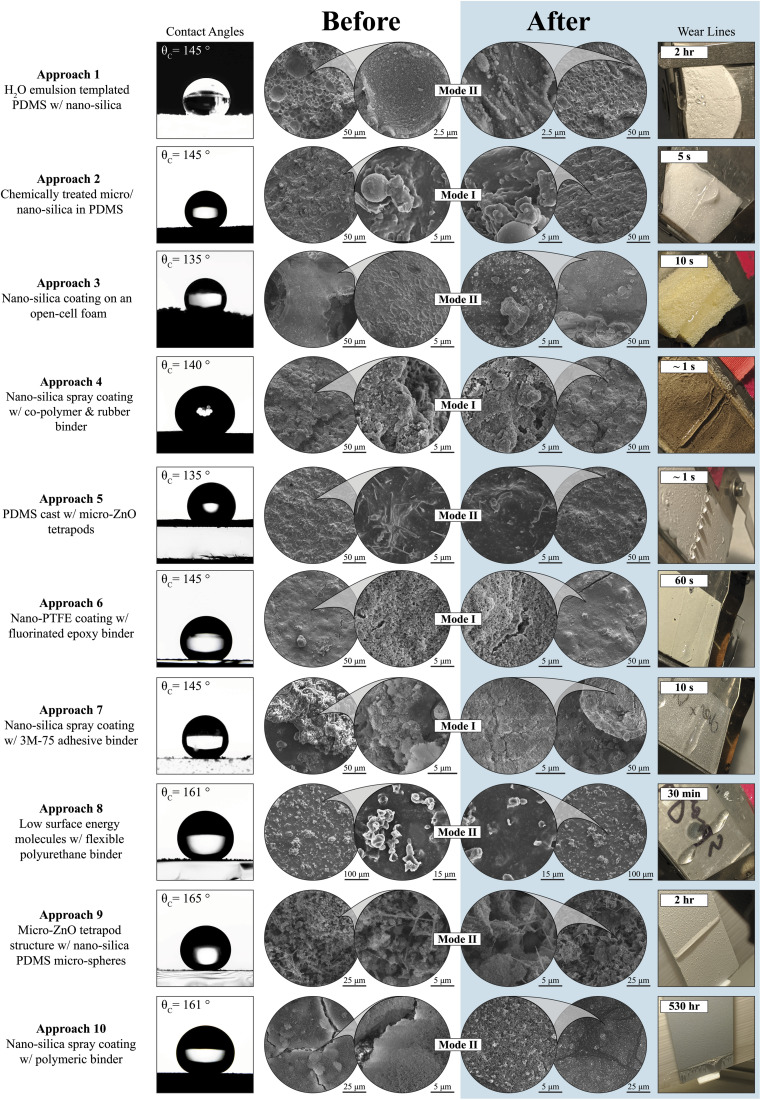
Overview of the synthesis results and the respective RDI testing results for previously reported coatings.^9–16^ Each row represents a distinct synthesis procedure to arrive at a superhydrophobic coating. Columns 1–3 are microstructural and hydrophobic performance results before RDI testing was conducted. Columns 4–6 are microstructural and RDI results. Column 6 presents that respective specimen at the time at which we consider the sample to be failed under RDI from our 16.5 mm disk at 5000-rpm test. Close-ups of columns 2–5 can be found with annotations in ESI.†

Approach 1: H_2_O emulsion templated PDMS w/nano-silica – a hydrophilic silica and water solution was incrementally added to un-cured polydimethylsiloxane (PDMS) and mixed thoroughly at each step. Eventually the emulsified water in PDMS mixture was cast and cured in an oven, and then heated to evaporate out the water. The resultant porous PDMS foam with nano-silica lined pores was sanded to increase the overall hierarchal roughness required for superhydrophobic properties. The procedure followed was based on Davis *et al.*^10^ Micrographs of the structures showed similar features to previous reports^10^ (ESI†). Of note, an unreported wt% limit was observed as the PDMS thickened with emulsions resulting in an upper limit of 51 wt% H_2_O/silica that could be added to the PDMS (previous reports cite having achieved up to 70 wt%). The coatings in this work featured structures exhibiting contact angles of 130° without silica, 136° with silica, and 145° with silica and the structure sanded with 240 grit sandpaper. These contact angles were similar to previous reports and the coatings were subjected to RDI tests. The approach 1 coating lasted an impressive ∼2 hours before wetting out. The before and after scanning electron microscopy (SEM) micrographs of Fig. 4 show that droplet impacts removed the silica from the PDMS pores in a clear representation of mode II hydrodynamic failure. Once enough of the loosely seated silica was pulled away from the PDMS, the coating was no longer able to avoid wet-out.

**Fig. 5:**
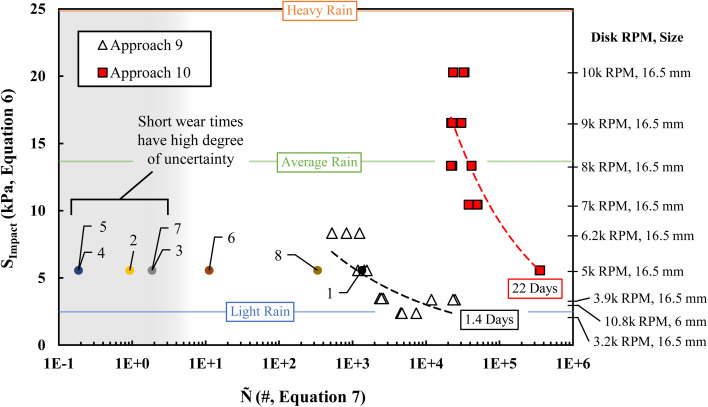
Droplet impacts per specific area to failure values for each of the 10 approaches are plotted against droplet impact stress values calculated according to eqn (6). The approach 9 coating demonstrates significant lifetimes that can reach 1.4 days. The approach 10 coating demonstrates remarkable lifetimes under RDI, reaching 22 days. For perspective, “light”, “average”, and “heavy” rain impact stresses are shown.^34^

Approach 2: chemically treated micro/nano-silica in PDMS – micro- and nano-silica spheres were chemically hydrophobized *via* a silanization surface treatment. After treatment, the particles were directly mixed into un-cured PDMS. The composite mixture was cast and then cured at high temperatures. When cooled, the silica-embedded PDMS was sanded to reveal micro/nano silica features in an attempt at superhydrophobicity. The procedure followed was informed by Zhang *et al.*, 2016.^13^ Of note, the approach was notably altered by substituting the arduous synthesis of silica particles with similar commercial hydrophobic silica (ESI†). The resultant mixtures were not self-leveling or superhydrophobic as previously reported.^13^ Approach 2 coatings cured well with no visible cracking and with good homogeneity. The coatings exhibited contact angles of 124° prior to sanding and 145° after sanding. This procedure demonstrated that the silanization of particles will increase particles' interaction with a PDMS binding matrix and result in improved hydrophobic properties.^13^ Sanding the coating created more complex hierarchical roughness to improve hydrophobic properties. Fig. 4 shows that droplet impacts altered the surface of the coating minimally and that mode I failure was dominant. The composite quickly wetted out, lasting only 5 seconds before failing under hydrodynamic wear.

Approach 3: nano-silica coating on an open-cell foam – chemically hydrophobized nano-silica was suspended in ethyl acetate. A piece of open-cell polyurethane (PU) foam was submerged in the silica suspension. The saturated foam was then dried. This resulted in a system of regular pores lined with nano-silica and with hydrophobic properties. The procedure followed was informed by Zhang *et al.*, 2017.^12^ Of note, the coating synthesized here included a different open cell PU foam than previous reports and employed commercial nano-silica instead of synthesizing in-house, resulting in a system with a smaller contact angle.^12^ The coating produced with approach 3 resulted in contact angles ranging from 130–140°. The silica–PDMS mixture failed to penetrate all the way through the PU sponge and only coated the outermost cells of the polyurethane structures. Since ethyl acetate did not dissolve or swell the PU foam used, interaction between the dissolved PDMS and suspended nano-silica was weak which led to the silica falling off when subjected to RDI. Fig. 4 shows that mode II failure was dominant. The system lasted 10 seconds before failure.

Approach 4: nano-silica spray coating w/co-polymer & rubber binder – acrylonitrile butadiene styrene (ABS) was dissolved in acetone and Plasti-Dip© was diluted with toluene. The two polymer suspensions were mixed together with hydrophobic nano-silica. The final mixture was then sprayed out onto a substrate and thermally cured in an effort to create a strongly bonded and slightly flexible superhydrophobic coating. The procedure followed was based on Milionis *et al.*^16^ Of note, the coatings synthesized in this work never entered the superhydrophobic range of previous reports of these coatings hitting 160° after thermal curing despite intensive effort. The reproduced suspensions of ABS and acetone and Plasti-Dip and toluene separated easily, with major clumps precipitating out. As a result, clumps of ABS and Plasti-Dip were left heterogeneously across the coated substrate. The areas with mostly nano-silica had contact angles of ∼140°. This lower contact angle compared with previous reports may be linked to the hydrophilicity of the ABS used, which may have provided instabilities in the created suspensions that can lead to zones of hydrophilicity developing within the coating. Incoming droplets would easily pin to hydrophilic zones – representing mode I hydrodynamic failure. Accordingly, the approach 4 coating immediately wetted out and showed no appreciable change in its surface after wear (Fig. 4).

Approach 5: PDMS cast w/micro-ZnO tetrapods – room temperature vulcanizing PDMS was diluted with ethyl acetate. ZnO tetrapods were suspended into the dilute elastomer. The composite mixture was then cast and set to dry into an elastic monolith with hydrophobic properties. The procedure followed was based on Yamauchi *et al.*^14^ The resultant samples showed great homogeneity upon curing. The tetrapods were well dispersed within the PDMS featuring a low packing density. Gaps between the tetrapod particles create the micro-roughness that led to hydrophobic properties. The reproduced samples had a contact angle of 135°. Though this value is lower than the previously reported values of 150°,^14^ our reproduced samples visually match the reported structures (ESI†). Mode II failure was dominant in this composite coating. Before and after SEM (Fig. 4) show a clear decrease in exposed ZnO tetrapod spines at the surface. As droplets impacted the coating, each impact fractured off an exposed portion of ZnO tetrapods rapidly reducing the already low hydrophobicity of this composite coating. This coating failed nearly immediately and stands as an example of when mode II failure is not enough to keep hydrophobicity when a coating is already not superhydrophobic (CA < 150°).

Approach 6: nano-PTFE coating w/fluorinated epoxy binder – a fluorinated epoxy was synthesized and then diluted in acetone. A suspension of nano-polytetrafluoroethylene (PTFE) particles was added to the epoxy mixture. After a complex sonication and mixing procedure, Krytox 1506 (fluorinated oil) was added prior to painting the solution onto substrates. The coatings were then thermally cured. The procedure followed was based on Peng *et al.*^15^ Of note, in the approach 6 coatings tested in this work, the suspensions quickly coarsened and became heterogeneous, and the fluorinated epoxy demonstrated a reduced contact angle relative to the non-fluorinated epoxy system. These coatings showed different behavior than previous work (*e.g.*, the coatings were not flexible and had lower contact angles of 145° *versus* previous reports of 158°, see discussion in ESI†) but were still subjected to RDI testing. Likely due to the lack of micro-roughness and the use of a hydrophilic binder, this coating wetted out rapidly in 60 seconds. Fig. 4 shows that mode I failure was dominant – the coating's surface was unchanged after wear testing.

Approach 7: nano-silica spray coating w/3M-75 adhesive binder – hydrophobic nano-silica was suspended into ethanol. In alternating order, a light coat of 3M-75 adhesive was sprayed followed by a spray coating of the silica–ethanol suspension. After multiple layers were applied, the coating was then set to dry at room temperature to evaporate the remaining solvents and reveal a hydrophobic system. The procedure followed was informed by Chen *et al.*^9^ Of note, the coatings tested in this work were produced with some alterations to the formulation and to the spraying techniques to improve achieved contact angle results, though previous reported contact angles of 159° were not attained despite intensive effort. Approach 7 coatings produced exhibited contact angles of 145° and were subjected to RDI testing. When investigated with SEM, the micrographs showed nano-roughness that was largely left unchanged after successive droplet impacts (Fig. 4). This coating wetted out in 10 seconds with a mode I failure occurring.

Approach 8: low surface energy molecules w/flexible polyurethane binder – a hydrophobic polyester binding material was diluted with chloroform. Octa-isobutyl POSS (IB-POSS), a low-surface energy molecule, was then added to the diluted polyester. The resultant mixture was then sprayed onto substrates and thermally cured. This combination of macro-molecules and slight miscibility between binder and solvent lead to superhydrophobic properties. The procedure followed was as described by Golovin *et al.*^11^ Coatings with approach 8 resulted in average contact angles of 161° similar to previous reports.^11^ Superhydrophobicity was achieved by using the slight phase separation between Desmophen and chloroform to create micro roughness on the coating's surface. The IB-POSS molecules acted as a means of chemically lowering the coating's surface energy but did not offer an increase to the coating's nano-roughness (Fig. 4). The samples could be sanded as reported and were found to maintain hydrophobic properties. The coating lasted 30 minutes under RDI before complete wet-out. Fig. 4 shows that some Desmophen microstructures were removed during wear testing, exhibiting that mode II failure was occurring.

Approach 9: micro-ZnO structure w/nano-silica PDMS micro-spheres – briefly, PDMS was chemically silanized and emulsified into an apolar solvent. The PDMS emulsions were then stabilized with chemically hydrophobic nano-silica particles. In addition to nano-silica, hydrophobic ZnO tetrapods were also suspended into the solvent mixture. The final suspension was then sprayed onto substrates and thermally cured. This procedure was informed from the apparent design principles of approaches 1–8 that led to longer wear lifetimes as discussed below.

The coating had an average contact angle of ∼165°. The multi-particle-system (MPS) was observed pre- and post-wear *via* SEM. The fresh sample had hierarchal roughness (Fig. 4). There was a macro-structure of ZnO tetrapods held together by small amounts of PDMS. This acted as the macro-roughness for the coating and as a structural system for which the micro-roughness could cling to. The micro-roughness of the hydrophobic system was created from nano-silica stabilized PDMS emulsions. These ∼2 µm micro-spheres, seen in Fig. 4, provided the complex nano-/micro-roughness required for the coating to be superhydrophobic.^1,31^ This superhydrophobic coating was powdery and could be rubbed away with mechanical wear. However, the delicate nature of this coating exhibited wear in a self-similar fashion under RDI.

The worn area of the MPS maintained most of the ZnO–PDMS macro-structure but had lost almost all the silica–PDMS micro-spheres (Fig. 4). Likely, each time a droplet impacted the MPS, a nominal amount of material was removed from the coating. Eventually, the majority of the exposed micro-spheres were removed and the coating was no longer superhydrophobic – showing mode II hydrodynamic failure. Under the test conditions chosen for approach 9 (Fig. 5), there was no regime of immediate wet-out observed. This coating lasted 2 hours for the 16.5 mm disk at 5000-rpm test and lasted 1.4 days under the 6 mm disk, 10 800-rpm test.

Approach 10: nano-silica spray coating w/polymeric binder – for this coating, a commercial superhydrophobic coating NeverWet Multi-Surface (Rust-Oleum, Illinois, USA) was used that comprises of two parts: a primer layer of dissolved polymer binder and a topcoat of suspended hydrophobic nano-silica. Both layers were applied *via* spray coating in accordance with the published directions from Rust-oleum.^32^ From a distance of 16 cm, the basecoat/primer layer was sprayed onto a glass substrate for a total of 2 coats. After 30 minutes, the following nano-silica/topcoat was sprayed at a similar distance for a total of 3 coats. The two-part coating was left to dry over night before testing. These samples had an average contact angle of ∼161°, which agreed with the published values ranging from 160–175°.^33^ The primer layer was slightly ductile and had good adhesion on most surfaces. The silica layer appeared to be binder-less. The solvent used to suspend the silica most likely was used to dissolve the primer coat material upon spraying, thus allowing some silica to embed and adhere to the primer coat after solvent evaporation. This adhesion method did pose as a weakness to abrasive wear; for, light rubbing on the coating would remove most of the silica layer in a similar fashion to approach 9.

The approach 10 coating was observed pre- and post-wear under SEM. The pristine structure showed an initial layer of binding primer, with a top-coat of nano-silica. The primer coat was not even and dried with clumps that acted as macro-roughness for the silica top-coat to build off of. The silica top-layer was densely packed with multiple cracks caused by solvent evaporation as the top-coat dried (Fig. 4). The two coats together created the micro and nano roughness needed to have hierarchal features that lead to superhydrophobicity.

The worn surface of the approach 10 coating showed silica-bare primer coat macro-features and lower densities of silica elsewhere throughout the worn surface. There were regular pits from which the silica that was in contact with primer was removed (Fig. 4). Based on the observed microscopy pre- and post-wear, the approach 10 coating likely exhibits mode II hydrodynamic failure. Each time a droplet impacts the approach 10 coating, an amount of silica was removed from the top-coat. After some time, there was a critical density of silica that was reached and determined whether droplets stuck and wetted out the underlying primer coat. The approach 10 coating lasted an astounding 530 hours under the 16.5 mm disk at 5000-rpm test.

## 4 Discussion

### 4.1 RDI performance of tested coatings

Many of the reproduced coatings described above did not achieve the same wetting characteristics as of their published works. These discrepancies are largely related to material and method alterations along with some reproducibility issues with the published procedures; all of which, are detailed in ESI.† A common measure for hydrophobic property resilience in the referenced papers above was abrasive wear.^9–16^ This choice of wear was good for simulating the wear and tear experienced in rough environments, but does not appropriately characterize the overall hydrophobic strength of the given coating. As shown in approaches 1 and 2, some methods to produce hydrophobicity rely on sanding as a final step to create hierarchical roughness and expose fresh apolar chemistry.^10,13^ Following such procedures with an abrasive wear test will not provide a clear indication as to how durable the hydrophobic properties of the coatings are – further characterization methods are needed.

The outcomes of the eight reported coatings are shown in Fig. 4. All samples were subjected to a standard test of a 16.5 mm disk spinning at 5000-rpm. Across all eight recreated works, no protocol produced a hydrophobic coating that was robust to RDI beyond 2 hours. The two reproduced coatings that lasted the longest, approach 1 and 8, exhibited mode II failure. These results are all graphically displayed in Fig. 4 along with the test results of approach 9 and approach 10. Consistent characteristics of the more successful coatings include structural hierarchy, chemical apolarity, and mode II dominant failure. These are further discussed in Section 4.3.

Fig. 5 shows the modeled droplet impact stress, *S*_Impact_, for each test condition against the number of droplet impacts per specific area to failure, *Ñ*. *Ñ*, is defined by the product of the droplets created per second, *n*, the percent of droplets that will hit the sample's surface, *p*, and the ratio of the droplet impact area of a single droplet to the sample wear area, *A*_w_, with time, *t*. Further definition of these terms can be found in ESI.†7
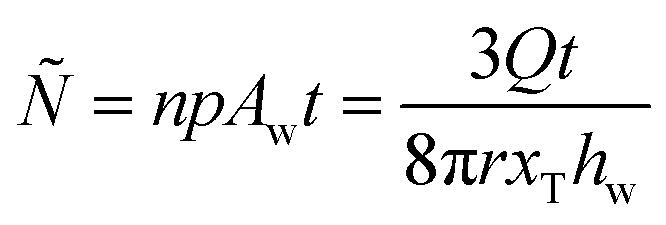
Here, *Q* is the flow rate (ml s^−1^), *x*_T_ is the sum of the radius of the hydrophilic disk and the distance from the nearest edge of the disk to the sample (see Fig. 2), and *h*_w_ is the wear band height measured after the RDI test with ImageJ.

Above ∼15 kPa, approach 10 begins to fail consistently after ∼2 × 10^4^ droplet impacts per specific area (Fig. 5). This demonstrates a regime where the coating starts to act heterogeneously. With a slope nearing infinity, a superhydrophobic coating would continue to fail spontaneously after a certain amount of droplet impacts; the hydrophobic structures would fail/or fall off all at once. Bellow 15 kPa, approach 10 develops a slope and start to exhibit “self-similar” wear characteristics. Wherein, approach 10 more evenly loses hydrophobic components over time. For the same 16.5 mm disk at 5000-rpm test, approach 10 was able to last an average of 530 hours, where approach 9 was only able to last an average of 2 hours. This ∼260× difference is sobering but is a critically fair comparison, proving that most academically reported self-similar superhydrophobic coatings likely have a long way to go to handle even RDI. Still the approach 9 coating offers some promise toward higher resiliency discussed in the next section.

### 4.2 Modeling RDI testing

The graph in Fig. 5 is similar in nature to the S/N curves used to describe the mechanisms of fatigue in materials subjected to cyclic stress.^35,36^ For the hydrodynamic wear studied here, the cyclic stress is provided by each instance of droplet impact. Hydrophobic materials more resilient to wet-out will be farther to the right on the chart. More resilient coatings (coatings that fatigue slower) will have a shallower slope. The trend lines present for both approaches 9 and 10 are power-law fits similar to mechanical fatigue power laws in S/N curves. These power law relationships can be used to estimate the time to failure under specified droplet impact conditions as expressed by the following equation:

8 *S*_Impact_ = *HÑ*^−*Σ*^

Here, *Σ* characterizes the resiliency of the hydrophobic coating. *Σ* values closer to zero suggest that a coating is more resilient and will not fatigue as quickly against hydrodynamic wear – demonstrating that the coating is either wearing more evenly (more self-similar) or not wearing at all (fatigue limit). Further, *H* in eqn (8) represents the ‘hydrophobic strength’ of a given coating. When *Ñ* ∼ 1, then a single droplet has sufficient impact stress to effectively neutralize the hydrophobicity of the coating and cause wet-out. Some of the approaches described above were led to failure even before *Ñ* ∼ 1. This shows that mode I failure, pinning, can occur below *Ñ* = 1, suggesting that those approaches were tested well above their hydrophobic strength. In Fig. 5, approach 10 (*H* = 751.8) has a far higher hydrophobic strength compared to approach 9, with a *H* = 35.12. Instead, approach 9 demonstrated a higher resiliency against fatigue caused from hydrodynamic wear with a *Σ* = 0.283, whereas approach 10 has a *Σ* = 0.401.

### 4.3 Trends in structure–property-performance for coatings subjected to RDI

The more resilient of the ten approaches shared three key attributes: (A1) apolar chemistry, (A2) hierarchical topography, and (A3) gradual spallation. Attributes (A1) and (A2) help avoid immediate failure under RDI – mode I failure, while attribute (A3) is consistent with self-similar wear characteristics and enables slow rate kinetics (long lifetimes) for mode II failure.

- Approach 1 exhibited all three key attributes (A1, A2, A3) and exhibited impressive performance. The composite PDMS foam structure provided apolar surface chemistry and an underlayment of microstructure for the adsorbed nano-silica particles. The spallation rate in which the nano-silica particles were removed under each droplet impact during RDI was also slow.

- Approach 2 exhibited only two key attributes (A1, A2) and was quickly wetted. The system's combination of PDMS and silanized soda-lime glass spheres provided both apolar chemistry and microstructural roughness. However, these components were not slowly removed during RDI, possibly leading to fast degradation of the apolar chemistry. Allowing droplet impingement and quick failure.

- Approach 3 exhibited only two key attributes (A1, A2) with a high-rate mode II wear. The foam used was a hydrophilic polyurethane which provided microstructural roughness, but also greatly reduced the amount of apolar chemistry within the composite. This system's hydrophobized nano-silica provided apolar chemistry and nano-scale roughness. Unfortunately, the bond between the nano-silica and the PU foam was weak and had a fast spallation rate, being removed in large sections with each droplet impact.

- Approach 4 exhibited only two key attributes (∼A1, A2) with no spallation occurring. The ABS and rubber binding layer was hydrophilic and did not provide any apolar chemistry. The added hydrophobic nano-silica provided apolar chemistry and hierarchical roughness but were not removed during RDI. Likely, the apolar chemistry was quickly degraded as the nano-silica remained embedded and mode I failure occurred.

- Approach 5 exhibited only two key attributes (A1, A2) with a high rate of spallation leading to an early failure. The PDMS matrix provided apolar chemistry while the ZnO-tetrapods provided microstructural roughness; however, the spallation of ZnO spines was not gradual and resulted in a short RDI test time.

- Approach 6 exhibited only two key attributes (∼A1, A2) and wetted out quickly. Though this system had apolar chemistry provided by the nano-PTFE particles, the hydrophilic epoxy binder allowed some water to remain pinned to the surface with each droplet impact. The particles did provide hierarchical roughness, and there was no spallation of the PTFE from the epoxy matrix.

- Approach 7 exhibited two key attributes (A1, A2) with no spallation occurring. The layered hydrophilic binder with hydrophobic nano-silica provided the apolar chemistry and hierarchical topography, but the particles were too bound to the binder and were not removed with droplet impacts.

- Approach 8 exhibited all three attributes (A1, A2, A3) and showcased impressive performance. The apolar chemistry was provided by the added IB-POSS molecules. A combination of the molecules and the binding matrix created hierarchical topology. During RDI each droplet impact led to the spallation of some IB-POSS structures at a slow degradation rate.

- Approach 9 exhibited all three attributes (A1, A2, A3) and displayed impressive performance. All components featured apolar chemistry. The coating also had high amounts of hierarchical roughness. Finally, the spallation of the nano-silica PDMS microspheres was gradual enough to extend the life of the coating under RDI.

- Approach 10 exhibited all three attributes (A1, A2, A3) and demonstrated impressive performance. The combination of a hydrophilic sublayer with micro-structure and hydrophobic nano-silica created apolar chemistry with hierarchical surface roughness. The weakly bound nano-silica particles were removed incrementally by droplet impacts resulting in very gradual spallation during RDI and extremely long lifetimes.

Across all ten coatings, the coatings that feature all three attributes (A1, A2, A3) featured more impressive coating lifetimes. Coatings that were apolar, hierarchical, and exhibited gradual spallation (characteristic of self-similar materials) performed better under RDI. This commonality suggests that these attributes are key for successful design of superhydrophobic coatings for droplet impact applications such as materials subjected to rainfall.

## 5 Conclusion

Self-similar superhydrophobic materials represent a promising and important field for creating self-cleaning, anti-bacterial, and anti-viral coatings. The ability to quantifiably measure how one coating performs relative to others is of vital importance to ensure fair and forward movement in this materials space. Droplet impact is a critical source of wear in superhydrophobic applications and cannot be ignored when considering such material systems. In this paper, we offer a method for characterizing hydrodynamic wear of self-similar superhydrophobic materials using RDI created from spinning disks. We developed an analytical model that accurately estimates the average droplet sizes created from the spinning disk in our setup. The model provides predictions on the impact stresses that sprayed droplets will apply to a coating during RDI testing. A total of 10 different approaches were tested using this RDI testing set-up. Many of the approaches tested did not last longer than 30 minutes, with some notable exceptions. Approach 9 lasted 1.4 days and approach 10 lasted 22 days. Both approach 9 and 10 can be characterized using power-law relationships like that of mechanical fatigue functions. Such a characterization method can provide an additional quantitative metric for the performance of hydrophobic coatings. Bringing quantitative metrics to how self-similar superhydrophobic coatings wear under rapid droplet impact will help measure success and drive this field forward.

## 6 Materials and methods

### 6.1 RDI wear test fabrication

In the contained environment of the test apparatus, a set of three samples were secured such that each sample was 35 mm and tangent from the edge of the droplet creation disk. This distance of 35 mm was chosen to ensure that the effects of gravity and drag to the droplet velocity vector were minimal. Water was fed *via* a Decdeal 12 V 5 W submersible water pump through a ∼200 µm stainless steel dispensing needle (McMaster-Carr) onto a porous cellulose sponge cloth (Swedish Wholesale) that covered the surface of a spinning disk. The cellulose sponge was used to ensure an even dispersion of water across the entire rotating disk. A 16.5 mm disk was spun by a XXD 1000 kV A2212 brushless motor and a 6 mm disk was spun by a FPVDrone 1104 7500 kV brushless motor.

### 6.2 RDI wear test methodology

Centrifugal force drove the water to build up at the edge of the spinning disk eventually overcoming surface tension and generating droplets that left the spinning disk at the tangential velocity and in the tangential direction toward the superhydrophobic coatings. On impact, droplets can either pin to or bounce off of a surface.^25^ Droplets that pin to a surface define mode I failure, where droplets are able to overpower hydrophobic forces and lead to rapid wet-out. Droplets that bounce off of a surface define mode II failure, where droplets remove an amount of material with each impact and more slowly lead to failure. Our determination of failure is a wholistic view of these two modes and was determined when the droplet impact region was saturated with enough water to create a visible bead across the width of the sample. A time-lapse camera was used to capture the wear tests to determine the “time to wetting” failure of each sample. All coatings were subjected to a baseline RDI test. The two highest performing coatings were subjected to varied intensities as described.

### 6.3 Contact angle measurements

Contact angles of pristine samples were measured *via* a telescope-goniometer^37^ to determine the angle tangent to a water droplet's contact with a given substrate.^37–40^ All contact angle images were captured *via* the same telescoping camera and were subsequently measured using the image analysis software ImageJ (NIH freeware). Lighting for each image was provided by a diffused light shadow box to avoid optical artifacts. The size of the droplets was kept to ∼9 mg of deionized water to minimize the effect of gravity on the resultant droplet shape. Of note, contact angles during and after RDI testing were not presently attainable. The wear area widths of RDI tested coatings were 2–4 mm, relatively heterogeneous, and had concave geometries, which made observation with a goniometer troublesome and inaccurate.

### 6.4 Scanning electron microscopy

Micrographs were obtained from a Hitachi S-4800 scanning electron microscope. Micrographs of samples pre and post wear were collected to investigate wear mechanisms and coating's self-similarities.

### 6.5 Synthesis of approach 9 coating

Sylgard 182 PDMS, mixed at a 10 : 1 weight ratio of part A to part B, was massed into a speed-mixing cup. 10 wt% trimethoxysilane (TMOS) was added to the PDMS components. A total of 70 wt% particles were added to the TMOS–PDMS mass: 60 wt% 10 µm titanate treated hydrophobic ZnO tetrapods (Dreytek) and 10 wt% 300 nm Aerosil R972 DDS treated hydrophobic nano silica. The particle/binder mixture was diluted to a 1 : 11 solids to solvent weight ratio with Vertrel XF. The suspension was then speed-mixed for 1 minute at 2500-rpm with a DAC 150.1 FVZ-K SpeedMixer (FlackTek, Inc.) and then again for one minute at 3500-rpm prior to spraying. The mixed suspension was filtered through a 300 µm filter to limit potential paint sprayer clogging. The solution was then sprayed through a 0.8 mm nozzle at 50 psi from a distance of 150 mm for a total of 5 coats. In this case, each coat was a striated horizontal motion up or down the substrate. The average coating thickness was ∼100 µm. After spray-coating, the samples were off-gassed in a fume hood for at least 30 minutes before curing the PDMS binder in a 100 °C oven over night.

## Conflicts of interest

There are no conflicts to declare.

## Supplementary Material

RA-013-D3RA00700F-s001
