# Supplementary material for: Understanding hydrodynamic wear in self-similar superhydrophobic coatings subjected to rapid droplet impacts
Source: RSC Adv. 2023 Apr 19;13(17):11356–67. doi: 10.1039/d3ra00700f (PMC10116186; doi:10.1039/d3ra00700f)
Supplement: RA-013-D3RA00700F-s001 [file RA-013-D3RA00700F-s001.pdf]

Electronic Supplementary Material (ESI) for RSC Advances.
This journal is © The Royal Society of Chemistry 2023

Title: Understanding Hydrodynamic Wear in Self-Similar Superhydrophobic Coatings Subjected to Rapid Droplet Impacts

Daniel J. Braconnier[1], Terence Davidovits[2], Randall M. Erb[1, *]

[1]Department of Mechanical and Industrial Engineering, Northeastern University, 360 Huntington Avenue, Boston, MA 02115, USA.

[2]Thermobionics LLC, Boston, MA, USA.

[*]r.erb@northeastern.edu

Supplementary Information

Velocity of a droplet affected by drag

The velocity of the droplet over time can be predicted as:

$$v = \frac{1}{(\omega R)^{-1} + \frac{3C_d\rho_1}{8\rho_2 r}t} \qquad (A).$$

$$a = \frac{-C_d\rho_1\pi r^2}{2m}v^2 = \frac{-3C_d\rho_1}{8\rho_2 r}v^2$$

$$\frac{dv}{dt} = A_1 v^2$$

$$\int \frac{dv}{A_1 v^2} = \int dt \ \rightarrow \ \frac{-1}{A_1}\frac{1}{v} + \frac{C_1}{A_1} = t + C_2$$

$$\frac{1}{v} = -A_1 t - A_1 C_2 + C_1$$

Where: t is time (s), $A_1 = \frac{-3C_d\rho_1}{8\rho_2 r}$, and C_1 , C_2 are integration constants.

Given.

$$\frac{1}{v_T} = -A_1 C_2 + C_1$$

$$\therefore \frac{1}{v} = -A_1 t + \frac{1}{v_T}$$

Velocity was then determined in terms of distance (x) rather than time, to assess whether or not drag would have a measurable affect on droplet travel in the later described hydrodynamic wear tests. Integrating **Equation A** with respect to time, gave the time required for a droplet to travel a distance x (**Equation B**).

$$\int v\,dt = \frac{8\rho_2 \ln(|3C_d\rho_1\omega Rt + 8\rho_2 r|)\,r}{3C_d\rho_1} + C = x \qquad (B).$$

Where: C is an integration constant.

Integration constant, C, was determined by setting the initial condition at $x = 0\,, t = 0$ in **Equation B** and gave rise to **Equation C**.

$$C = \frac{-8\rho_2 \ln(8|\rho_2 r|)\,r}{3C_d\rho_1}$$

$$\therefore x = \frac{8\rho_2 \ln(|3C_d\rho_1\omega Rt + 8\rho_2 r|)r}{3C_d\rho_1} - \frac{8\rho_2 \ln(8|\rho_2 r|)r}{3C_d\rho_1} \qquad (C).$$

By solving **Equation C** for time, the time, t^* at which a droplet hits the sample at distance x_T ($x_T = R + distance\ to\ sample\ \ [m]$) was determined to be (**Equation D**):

$$t^* = \frac{8\left(\rho_2 r e^{\frac{3C_d\rho_1 x_T}{8\rho_2 r}} - \rho_2 r\right)}{3C_d\rho_1\omega R} \qquad (D).$$

Substituting **Equation D** into **Equation A** simplifies to **Equation E**, and is shown below:

$$v^* = \omega R e^{\frac{-3C_d\rho_1 x_T}{8\rho_2 r}} \qquad (E).$$

Where: v^* is the droplet velocity at impact (m/s).

Effect of drag on the velocity of generated droplets from a spinning disk

The effect of drag on droplets was assessed from video footage where the disk's edge was in frame. Droplet velocities that could be associated with a known distance travelled from the disk were graphed against the modeled droplet velocity. For a droplet's velocity per distance travelled (affected by drag), the modeled droplet velocities showed a linear decrease in magnitude. Showing that for the droplet creation conditions we would be testing the drag coefficient could be considered independent and constant. **Figure S1** shows that the velocity of a given droplet is relatively constant over a distance of 10 cm. Thus, drag can be ignored for short distances tested over in this paper (C_d = 0, tangential velocity).

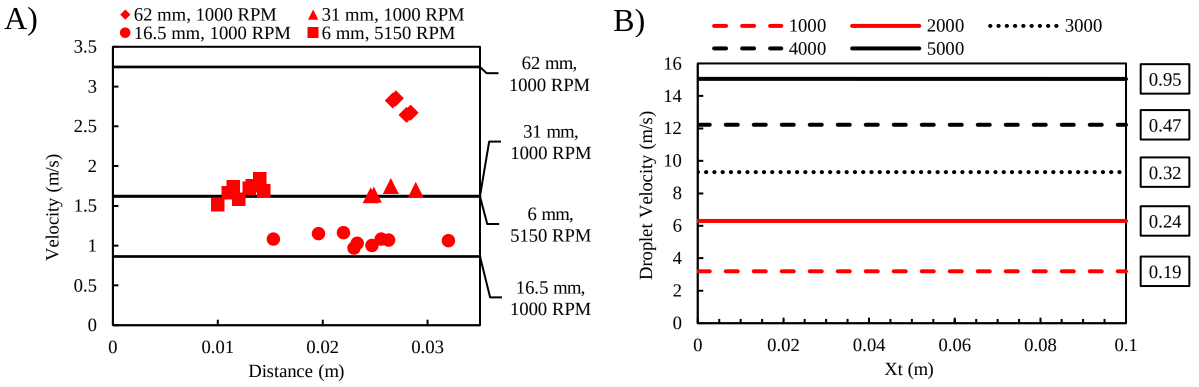

Figure S1: *(A) Droplet velocities at known distances changed negligibly. (B) With a C_d of 0.5, the velocities of droplets from a 62 mm disk are nearly constant over a 10 cm distance (modeled droplet diameters boxed).*

Approach 1: H_2O emulsion templated PDMS w/ nano-silica

The materials required for this process were Evonik Aerosil 300 hydrophilic nano-silica, Sylgard 182 polydimethylsiloxane (PDMS), and de-ionized H_2O (DI). The published procedure was followed with some minor changes: a Flacktek speedmixer was used instead of the described fixed RPM impeller [S1], a syringe was used instead of the referenced syringe pump, the use of a vacuum prior to curing the samples was disregarded as speed-mixing removes bubbles from mixtures [S2], and samples were baked at 100 °C instead of 180 °C to minimize cracking due to rapid evaporation of water emulsions.

2 g of hydrophilic nano-silica was suspended into 38 g of DI to create a 5 wt% nano-silica solution. The solution was then sonicated via tip sonication at an intensity of 1 for 12 minutes. Using a syringe, 0.25 ml of nano-silica solution was incrementally added to 20 g of PDMS. After each increment, the PDMS and nano-silica solution was speed-mixed for 45 seconds at 1000 - RPM. This process was repeated until 50 wt% of the silica solution had been emulsified into the PDMS. With speedmixer cup sealed to limit water evaporation, the sample emulsion was cured at 60 °C for 4 hours. Once cooled, the cured PDMS structure was removed from the speed-mixing cup and then baked at 100 °C for 5 days.

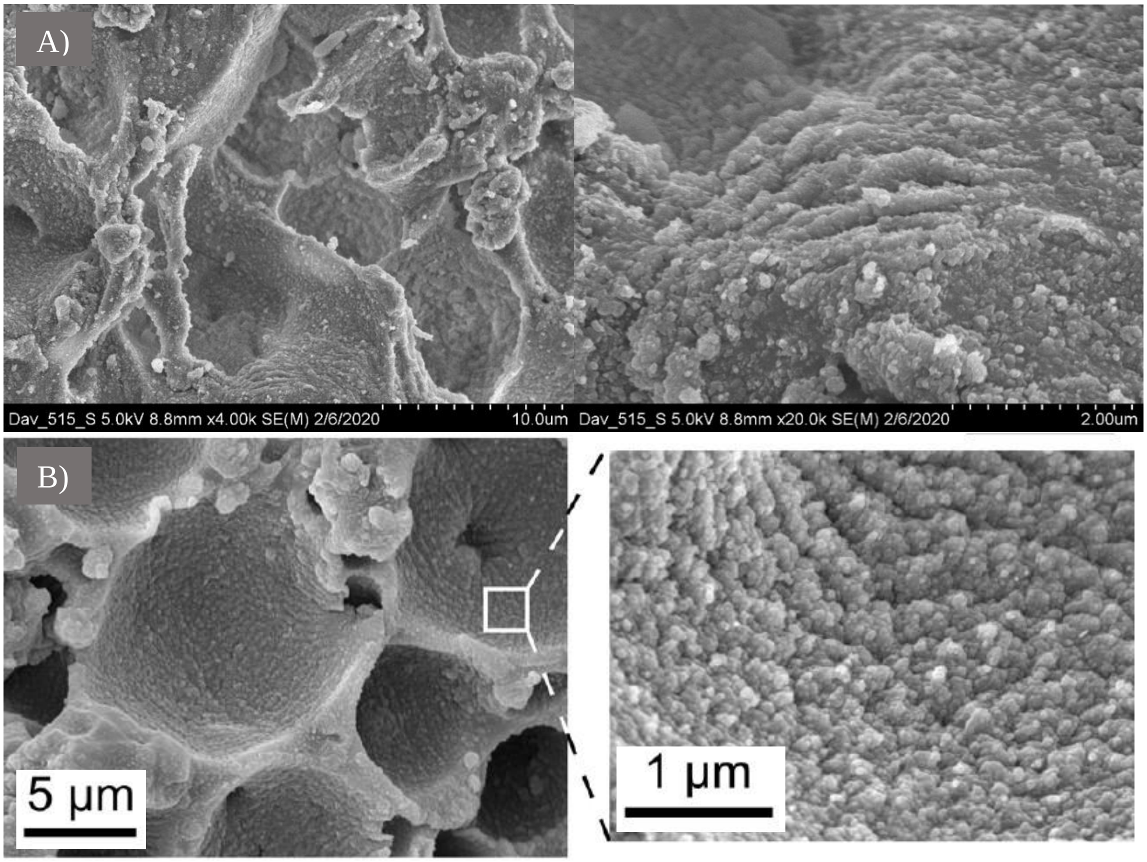

Figure S2: *The silica coating on the PDMS pore walls in the reproduced sample (A) is similar to the coating seen in samples created by Davis (B).*

Approach 2: Chemically treated micro/nano-silica in PDMS

Materials required for this procedure were: nano-silica, micro-silica spheres, silane, and Sylgard 184 PDMS. The published procedure was followed with some minor changes: Aerosil R972 hydrophobic nano-silica and Prizmalite P2015SL soda-lime 8-12 µm micro spheres were used instead of the published lab-made silica particles, and Gelest 98% 1,1,1,3,3,3-hexamethyldisilazane (HMDS) was used to silanize the soda-lime spheres.

Hydrophobic soda-lime micro spheres were created via silanization. A 1 wt% HMDS in H_2O solution was created and the soda-lime spheres were added to at a mass ratio of 1:5 silane to particles. In a polypropylene (PP) beaker, this mixture was magnetically stirred at 90 °C until all water had evaporated (~ 12 hours). Next, isopropyl alcohol (IPA) was added to rinse the silanized particles and stop any continuing reaction. The IPA was evaporated off in an oven set to 90 °C.

In a speed-mixing cup, 1.5 g of hydrophobic nano-silica, 1.7 g of hydrophobic soda-lime micro spheres, and 8 g of PDMS were speed-mixed together at 2500-RPM for 1 minute. The resultant mixture was spread out onto a glass slide and cured in an oven at 210 °C for 1 hour. Finally, the cure and cooled sample was sanded with 220 grit sandpaper to increase micro-roughness and expose embedded silica/soda-lime particles.

Approach 3: Nano-silica coating on an open-cell foam

The materials required for this procedure were: open-cell polyurethane (PU) foam (stock from manufacturer), lab-made silanized nano-silica, high-purity ethyl acetate (Sigma Aldrich), and polydimethylsiloxane. The published procedure was followed with some minor changes: Aerosil R9200 and R972 nano-silica were used instead of the lab-made silica, two-part PU foam was used to create the required open-cell structure, and Sylgard 182 PDMS was used.

0.18 g of hydrophobic nano-silica was dispersed in 60 g of ethyl acetate. 0.05 g of un-cured PDMS were the dissolved in the ethyl acetate solution. After all components were fully incorporated via magnetic stirring, a piece of PU foam was soaked in the mixture for 30 minutes. The soaked sponge was then baked in an oven at 110 °C for 1 hour to evaporate the ethyl acetate and cure the PDMS binder.

Approach 4: Nano-silica spray coating w/ co-polymer & rubber binder

Milionis used acrylonitrile butadiene styrene (ABS) pellets, Aerosil R812S HMDS treated nano-silica, Plasti-Dip ©, high-purity acetone, and toluene to create their published superhydrophobic spray coating. The published procedure was followed as written.

Three solutions were created: 5 wt% dissolved ABS pellets in acetone, 5 wt% nano-silica in acetone, and 50 wt% Plasti-Dip in toluene. A volume ratio of 5:2 ABS solution and silica solution was created. The Plasti-Dip solution was added to the ABS/silica mixture at a volume ratio of 1:7. The final mixture was then sprayed onto aluminum plate from a distance of 150 mm at a pressure of 30 psi through an airbrush. The resultant coating was then heated at 240 °C for 25 minutes to evaporate the solvents and slightly melt the ABS binder.

Approach 5: PDMS cast w/ micro-ZnO tetrapods

To replicate this published procedure, room temp vulcanization (RTV) PDMS, 10 um zinc-oxide (ZnO) tetrapods, and ethyl acetate were required [S3]. GE Clear RTV Silicone was used in place of the DOW HC2100 silicone.

1 g of RTV PDMS was dissolved in 6 ml of ethyl acetate and mixed for 1 minute via magnetic stirring. 1 g of ZnO tetrapods was then added to the dissolve PDMS solution. The particle loaded mixture was then painted out onto a substrate and set to dry for 1 week.

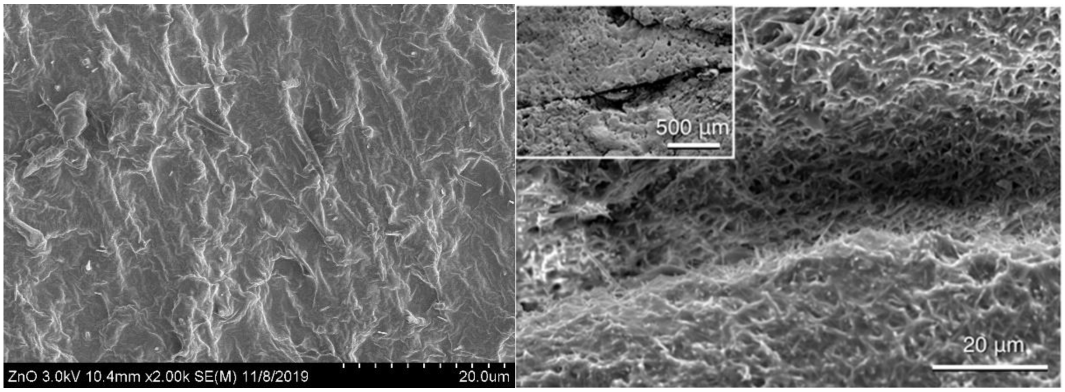

Figure S3*: The ZnO-tetrapod structure within the PDMS of the reproduced sample (left) is similar to the structures seen in samples created by Yamauchi (right).*

Approach 6: Nano-PTFE coating w/ fluorinated epoxy binder

The materials required were: diethylenetriamine (Sigma Aldrich), heptafluorobutyric acid (Sigma Aldrich), DI H_2O, Airstone 760E epoxy, high purity acetone, 250 nm PTFE particles, and Krytox 1506 oil. The PTFE particles were sourced from a different supplier than referenced by Peng [S4].

First a F-amine curing agent was created. 0.01 mol of diethylenetriamine was mixed into 10 ml of DI. Separately, 0.01 ml of heptafluorobutyric acid was stirred into 10 ml of DI. The diethylenetriamine mixture was stirred and brought to 100 °C on a magnetic hotplate. The acid mixture was added dropwise to the heated amine mixture. The reaction was run until all water had evaporated. Next a particle suspension was created. 2 g of epoxy resin were dissolved in 5 ml of acetone. Separately, 10.5 g of PTFE nano-particles were suspended in 30 ml of acetone. Both epoxy and PTFE mixtures were combined on a stir plate and then 0.3 g of Krytox 1506 was added to the solution. The final mixture was bath sonicated for 15 minutes. Finally, 1.5 g of F-amine curing agent was diluted with 10 ml of acetone. The f-amine acetone solution was then mixed with all of the created particle/epoxy suspension. This final mixture was bath sonicated. The created suspension was painted onto substrates and cured at 100 °C for 1 hour.

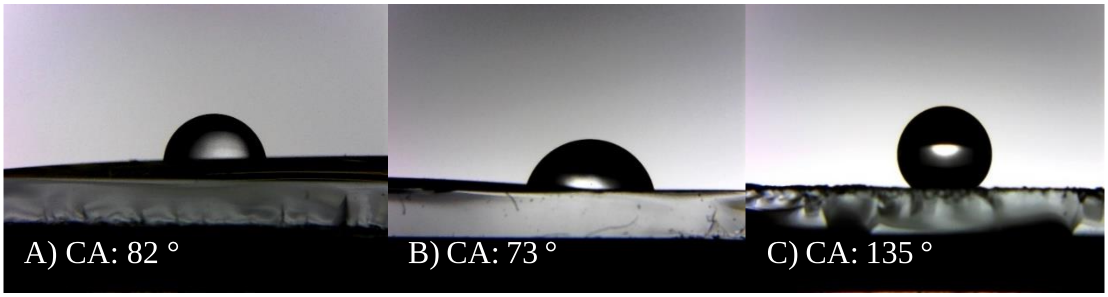

Figure S4: *The amine fluorination caused a decrease in the contact angle of the epoxy. (A) Epoxy, no fluorination (B) Epoxy, fluorinated (C) Fluorinated epoxy w/ PTFE particles.*

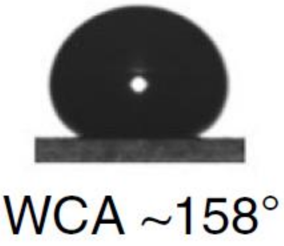

Figure S5: *Image from published Figure 1d [S1], showing a contact angle image of a sagging droplet. Our remeasured angle from this is closer to 148-150 °, than the reported 158 °.*

Approach 7: Nano-silica spray coating w/ 3M-75 adhesive binder

Materials required for this procedure were: Aerosil R972 hydrophobic nano-silica, 3M 75 spray adhesive, and ethanol [S5]. Aerosil R9200 hydrophobic nano-silica was used in our rendition of this procedure for its improved CA compared to R972 when used in the way described below.

A silica suspension in ethanol was create at a 1:19 ratio of silica to ethanol. To apply the coating to a substrate, an alternation of adhesive and silica spray was used. First a layer of 3M 75 was sprayed on a glass slide from a distance of 400 mm. Then a layer of silica/ethanol mixture was sprayed via airbrush from the same distance. These two steps were repeated to achieve a desired thickness. Afterwards, samples were dried for at least 5 minutes to evaporate the remaining solvents in the adhesive and the remaining ethanol. Spraying successive layers as in the published steps led to a coating that was mostly 3M adhesive and hydrophilic. Spraying only one layer of each step resulted in the best contact angle and hydrophobic properties.

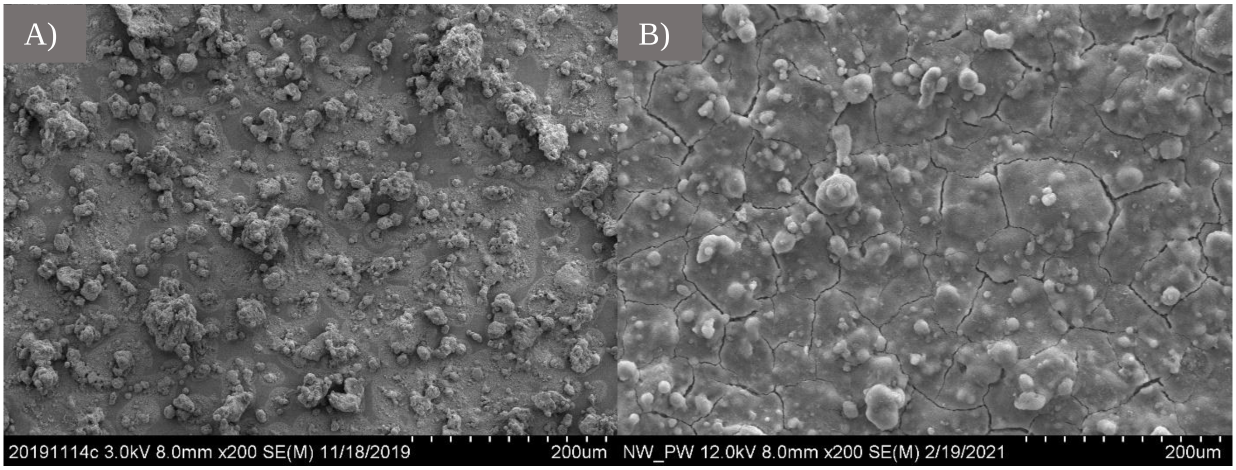

Figure S6: *The sprayed silica suspension (A) formed clumped structures that are comparable to the silica structures found in commercial NeverWet (B).*

Approach 8: Low surface energy molecules w/ flexible polyurethane binder

This procedure required Covestro Desmophen 670BA (a slightly branched hydroxyl-bearing polyester), Covestro Desmodur N3200 (aliphatic polyisocyanate), Octa-Isobutyl POSS (IB-POSS), and high purity chloroform. No material changes had to be made in the replication of this procedure.

Desmophen and Desmodur were mixed together at a mass ratio of 5:2 respectively. 30 wt% IB-POSS was added to the binder mixture. Chloroform was then added to the particle/binder mixture at a ratio of 15:1. The suspension was then mixed in a speedmixer at 2500-RPM for 1 minute, followed by a 3500-RPM mix for another minute. The mixed suspension was sprayed onto a substrate at 50 psi through a 0.8 mm spray nozzle. The coating was off-gassed for at least 30 minutes before it was placed in an 80 °C oven over night to cure the binding matrix.

Number of Cycles: Droplet impacts per specific area to failure

The total number of droplets created per second is given by:

$$n = \frac{Q}{V_r} = \frac{3Q}{4\pi r^3} \qquad (F).$$

Where: Q is flow rate (ml/s) and V_r is the volume of a single droplet.

The percentage of droplets that will travel to hit the sample's surface, p, is defined by the ratio of sample width, w, to the circumference of the droplet creation system (**Equation G**).

$$p = \frac{w}{2\pi x_T} \qquad (G).$$

The radius of the droplet creation system includes the radius of the hydrophilic disk and the distance from the nearest edge of the disk to the sample; given by, x_T.

The ratio of the droplet impact area of a single droplet to the sample wear area, A_w, was defined to account for changes in wear area due to slight changes in spray area observed when testing with different disk sizes. This value is given below:

$$A_w = \frac{\pi(r)^2}{w * h_w} \qquad (H).$$

Here, w is sample width and h_w is the wear band height. h_w was measured post-test with ImageJ.

Close-ups of Micrographs in Figure 4

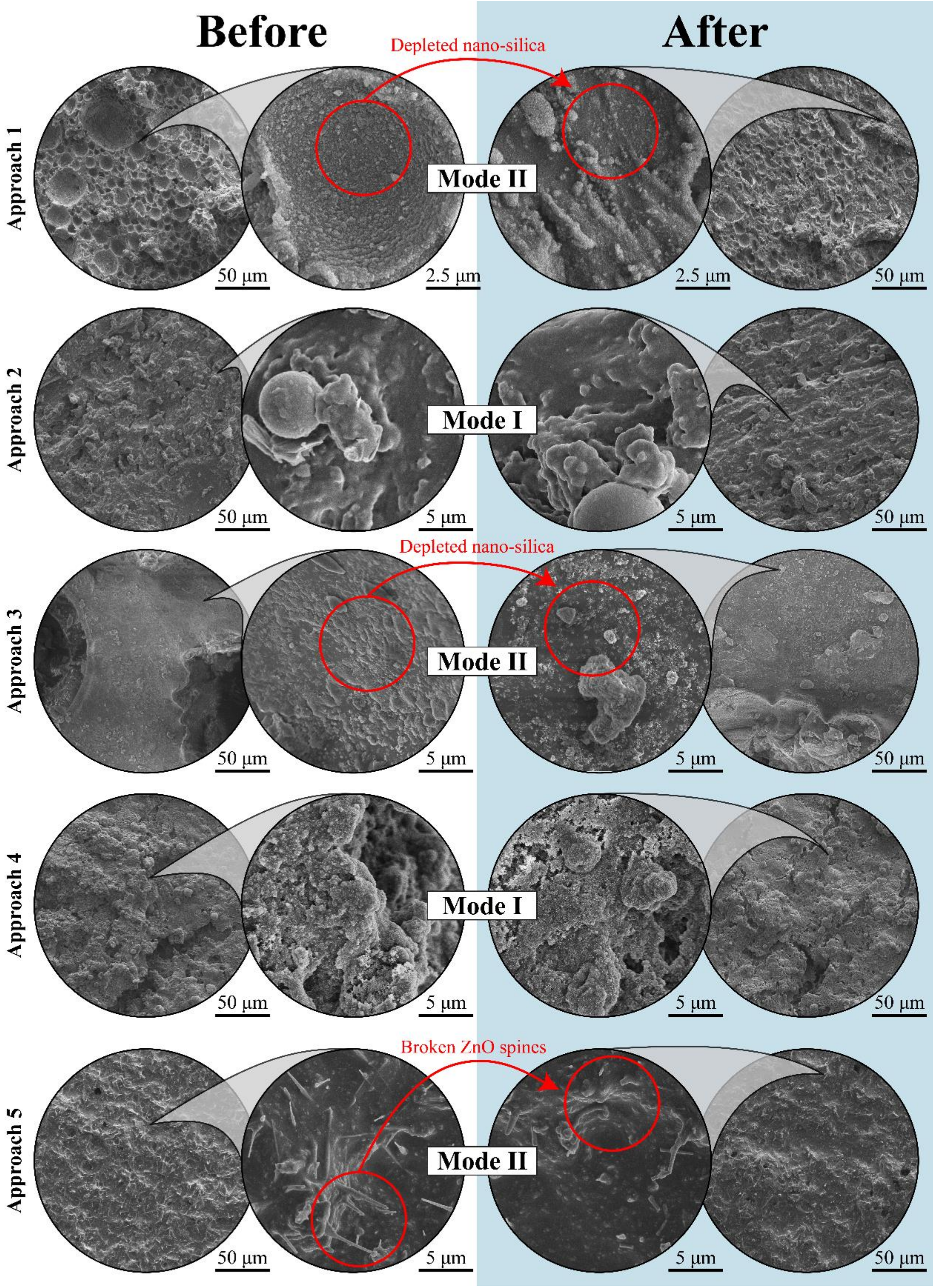

Figure S7: *Close-up micrographs from* ***Figure 4*** *of the main paper with annotations highlighting the evolution of microstructures during hydrodynamic wear.*

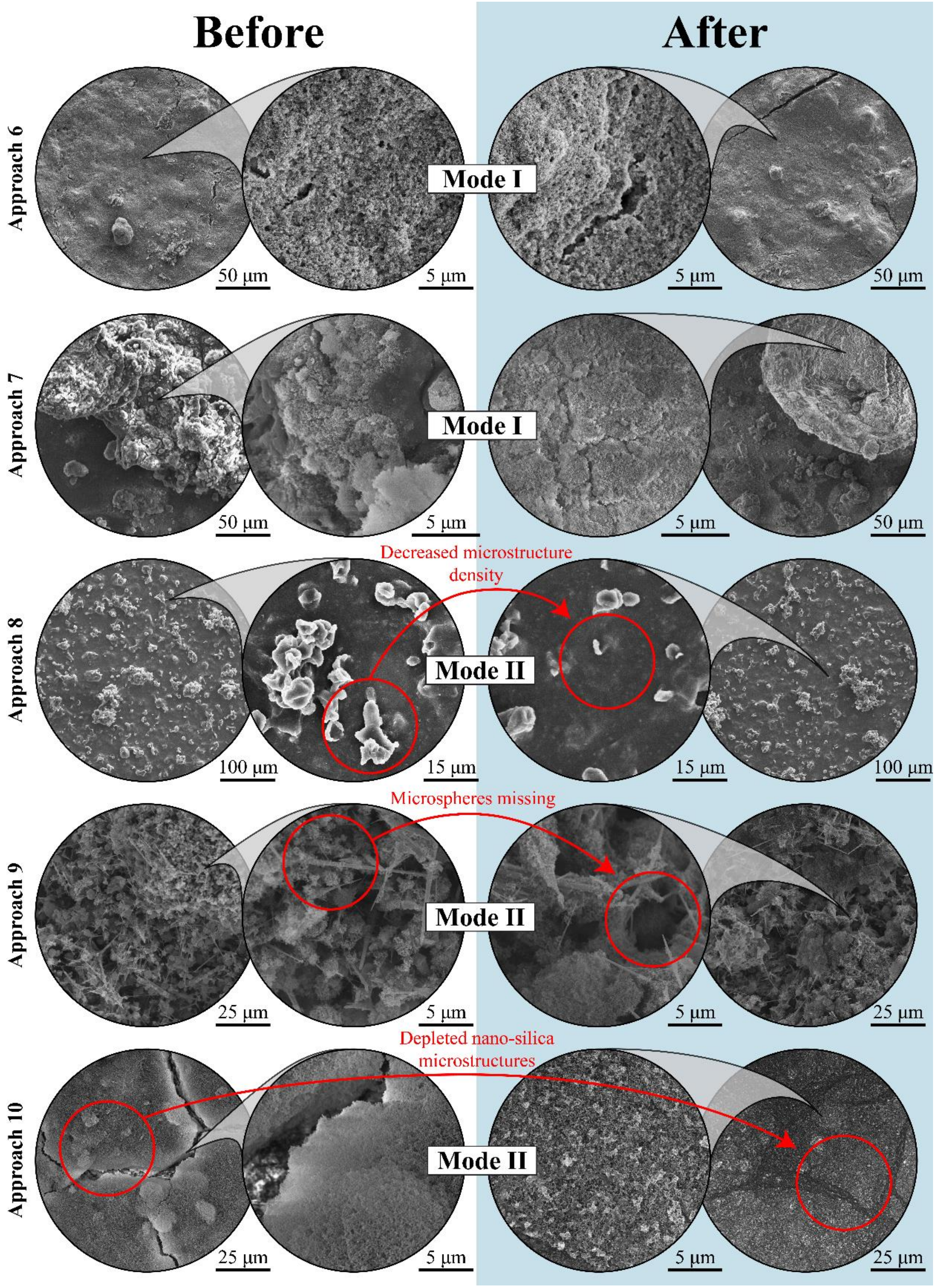

Figure S8: *Close-up micrographs from* ***Figure 4*** *of the main paper with annotations highlighting the evolution of microstructures during hydrodynamic wear.*

References

[S1] A. Davis, S. Surdo, G. Caputo, I. S. Bayer and A. Athanassiou, "Environmentally Benign Production of Stretchable and Robust Superhydrophobic Silicone Monoliths," *ACS Applied Materials and Interfaces*, vol. 10, pp. 2907-2917, 2018.

[S2] F. Speedmixer, "The Technology," Flacktek, [Online]. Available: https://speedmixer.com/the-technology/. [Accessed 7 10 2021].

[S3] Y. Yamauchi, M. Tenjimbayashi, S. Samitsu and M. Naito, "Durable and Flexible Superhydrophobic Materials: Abrasion/Scratching/Slicing/Droplet Impacting/Bending/Twisting-Tolerant Composite with Porcupinefish-Like Structure," *ACS Applied Materials and Interfaces*, vol. 11, pp. 32381-32389, 2019.

[S4] C. Peng, Z. Chen and M. K. Tiwari, "All-organic superhydrophobic coatings with mechanochemical robustness and liquid impalement resistance," *Nature Materials*, vol. 17, pp. 355-360, 2018.

[S5] C. Chen, D. Weng, S. Chen, A. Mahmood and J. Wang, "Development of Durable, Fluorine-free, and Transparent Superhydrophobic Surfaces for Oil/Water Separation," *ACS Omega*, vol. 4, pp. 6947-6954, 2019.
